# A Novel Self-Assembled Gel for Gastric Endoscopic Submucosal Dissection-Induced Ulcer: A Preclinical Study in a Porcine Model

**DOI:** 10.3389/fphar.2021.700387

**Published:** 2021-10-01

**Authors:** Meng Li, Haifeng Jin, Changpei Shi, Bin Lyu, Xiao Ying, Yuan Shi

**Affiliations:** Department of Gastroenterology, First Affiliated Hospital of Zhejiang Chinese Medical University, Hangzhou, China

**Keywords:** artificial ulcer, endoscopic submucosal dissection, gel, self-assembled, ulcer healing

## Abstract

Endoscopic submucosal dissection (ESD) is a routine procedure for the management of early gastrointestinal neoplasia, but it results in large ulcers. This study aims to examine the feasibility and effectiveness of a newly developed self-assembled gel on the ulcer healing process after ESD. Sixteen 2.0-cm (diameter) gastric ulcers were created by ESD in five pigs. All ulcers were randomized: control group (*n* = 7, routine ulcer management) and gel-treated group (*n* = 9). The gel was applied to cover the whole ulcer bed through the endoscope immediately after ESD. The feasibility of this endoscopic treatment modality was macroscopically accessed by endoscopy. The effectiveness was evaluated based on the ulcer area and histology changes at 14 days after ESD. The gel-treated group showed higher healing activity compared with the control ulcers on day 14 after ESD. The percentage of healing was higher for the gel-treated ulcers than in control ones (96.2 ± 2.2% vs. 91.9 ± 4.5%, *p* = 0.035). The extent of the new epithelium covering the ulcer was greater in the gel group than in controls. One delayed overt bleeding occurred in the control group (14.3%). This novel gel might promote the speed of ulcer healing after ESD, leading to higher epithelium formation.

## Introduction

Endoscopic submucosal dissection (ESD) is a standard procedure for the management of early gastric cancers (NCCN, 2020; Wang et al., 2015; Bennett et al., 2009). Compared with endoscopic mucosal resection (EMR), ESD allows *en bloc* resection of large lesions, provides a better histopathologic staging, and have a lower recurrence rate (Gotoda et al., 2006; Uraoka et al., 2009; Tanaka et al., 2015; Tanaka et al., 2020). On the other hand, the main disadvantage is that ESD usually results in a larger ulcer wound surface than EMR. It has been reported that an ESD-induced ulcer needs 8 weeks to heal (Kakushima et al., 2006a; Goto et al., 2011).

During the healing process, the patients will suffer from pain and be at risk of perforation and bleeding. Delayed bleeding is seen in 4.6–15.6% of the patients who undergo ESD for gastric lesions (Takizawa et al., 2008; Chung et al., 2009; Goto et al., 2012; Mochizuki et al., 2015). Nevertheless, artificial ulcer healing is relatively faster than that of a usual gastric ulcer, but it still requires about 8 weeks to heal (Goto et al., 2011). During the first 4 weeks after ESD, the ulcers are in a healing state; mucosal contraction results into a significant size reduction of the ulcer, then regenerative mucosa covers the mucosal defect, and the ulcer turns into the scarring stage with very few recurrences (Kakushima et al., 2006b). For the first 3 days after ESD, massive bleeding usually occurs (Goto et al., 2011). Hence, in the very early phase, it is critical to protect the ulcer floor from the attack of gastric or bile secretions to ensure that the regenerative epithelium completely covers the surgical defect and to avoid complications.

Various methods were tried to accelerate artificial ulcer healing or reduce post-ESD adverse events (Nishizawa et al., 2015; Maruoka et al., 2017; Ko et al., 2019). The mechanical protection of the ulcer bed protects the mucosal defect against gastric acid and bile secretions during early healing. Some researchers try to cover the ulcer bed with a polyglycolic acid sheet (PGAs) or bio-sheets (Kwon et al., 2015; Sakaguchi et al., 2015), but this strategy is expensive and time-consuming, and the success is somehow uncertain.

A novel gel was designed by our team. It is composed of a colloidal solution and a fixative solution. The colloidal solution is composed of seaweed polysaccharides and polylysine. The fixative solution contains calcium salts. The material polymerizes in an acidic environment like the stomach to cover the wound. It is biocompatible, non-pyrogen, and does not contain any ingredients of animal or human origin. The colloidal solution is applied to the gastric mucosal wound surface and then sprayed with the fixation solution. When gastric acid flows to the wound, the cross-linking effect of polylysine and seaweed polysaccharides occurs under the action of gastric acid, forming a protective film of seaweed polysaccharide-polylysine cross-linking complex. Covering an ulcer with the specific gel might promote the regeneration of the mucosa and speed the healing of the ulcer.

Therefore, this porcine study aimed to assess the feasibility and efficacy of this newly developed self-assembled gel on the ESD-induced ulcer healing process. The results could define the feasibility of its use as an endoscopic treatment modality after ESD and could allow the design of a clinical trial.

## Materials and Methods

### Study Design

This was a randomized controlled prospective animal study. The study was conducted at the Zhejiang Chinese Medical University. This study was approved by the Zhejiang Chinese Medical University of Medicine Institutional Animal Care and Use Committee (#HZYJ20191203).

### Animals

Five female mini pigs (strain, *Sus scrofa*; weight, 20–30 kg; age, 2–3 months) were used. Three or four 2.0-cm artificial ulcers were created in each pig by ESD. Each ulcer was randomized (using coin tossing) to the control group and the gel-treated group.

### ESD

A conventional endoscope was used to carry out ESD. The greater curvature of the stomach was selected. A GIF-Q260J endoscope (Olympus, Tokyo, Japan), a D-201 disposable distal attachment cap (Olympus), an EVIS 260 Spectrum system (Olympus Medical Systems), and an electrosurgical unit were used for ESD. A glycerin solution stained with indigo carmine was used to lift the submucosal layer by injection, an IT knife nano (KD-612Q) or Dual Knife (KD-650Q) electric knife (Olympus) was used to incise the mucosa circumferentially and perform the submucosal dissection. Hemostatic forceps (Coagrasper; Olympus Medical Systems) were used to coagulate any exposed vessels visible in the ulcer floor.

### Description of the Self-Assembled Gel

The novel gel consists of two components (colloid solution and fixative solution). The colloid solution consists of algal polysaccharides and polylysine. The fixative solution contains calcium. Both solutions are nontoxic, biocompatible, and proprietary. The two solutions are mixed and rapidly self-assemble into a solid film made of a complex network of polysaccharides and amino acids, which further solidifies in the presence of gastric acid. The network adheres to the ulcer floor through ionic interactions. For each ulcer, 20–30 ml of the colloid solution and 10–20 ml of the fixative solution are used to cover a 2-cm ulcer. The ulcer and surrounding area were cleaned. The colloid solution was sprayed on the ulcer surface through the endoscope. Then, the fixative solution was sprayed. The gel solidified by 3–5 min. The gel was required to cover the ulcer. In the control group, the ulcers were managed routinely.

### Data Collection

The surgical time was defined as the time starting with mucosal incision and finishing with the complete separation of the resected specimen. The injection time was measured separately. The speed of cutting was calculated as the resection area divided by the cutting time. Both the volume and the time required to apply colloid solution or fixative solution was calculated separately. Significant bleeding was considered if the need to use the Coagrasper forceps (Olympus) arose during the procedure.

### Follow-Up Endoscopic Examination

When the procedure was completed, repeat endoscopy was carried out on day 7 with the animal under anesthesia to assess the healing of the resection sites and the gel status. A residual congealing gel was defined as loosely or locally stacked white membranous film at the base of the untreated ulcer. The animals were examined carefully for hemorrhage and delayed perforation. Euthanasia was performed on day 14. The animals underwent a necropsy.

### Evaluation of Artificial Ulcer Healing

After ESD, the initial removed tissues were pinned to a rubber board and fixed in 10% neutral buffered formalin. 14 days after ESD, the harvested stomach from each pig was carefully cleaned, and the remaining ulcer sites were completed resected. Using the ImageJ 1.47 analysis system (National Institutes of Health, Bethesda, MD, USA), the area of the initial resected specimen and that of the remaining ulcer were measured and quantified. The proportion of residual ulcer was calculated as (ulcer area)/(area of initial ulcer). The healing proportion was calculated as 100—(percentage of residual area of ulcer).

### Histopathologic Evaluation

All the 14-days fixed lesions including the ulcer and adjacent normal tissue were taken along the long diameter for the histology examination. The non-epithelized tissue diameter (ND) was defined as the diameters on the long axis. Mucosal dissected tissue diameter (MD) was defined as the maximal length in a cross-section. The relative re-epithelized level of the wound (Wre) was described by

Wre=MD−ND/MD

### Statistical Analysis

The results are presented as means ± standard deviations and analyzed using Student’s t-test. Statistical significance was considered with two-sided *p*-values <0.05. All statistical analyses werfe performed using SPSS 16.0J for Windows (IBM, Armonk, NY, USA).

## Results

### Characteristics of the Ulcers

**FIGURE 1:**
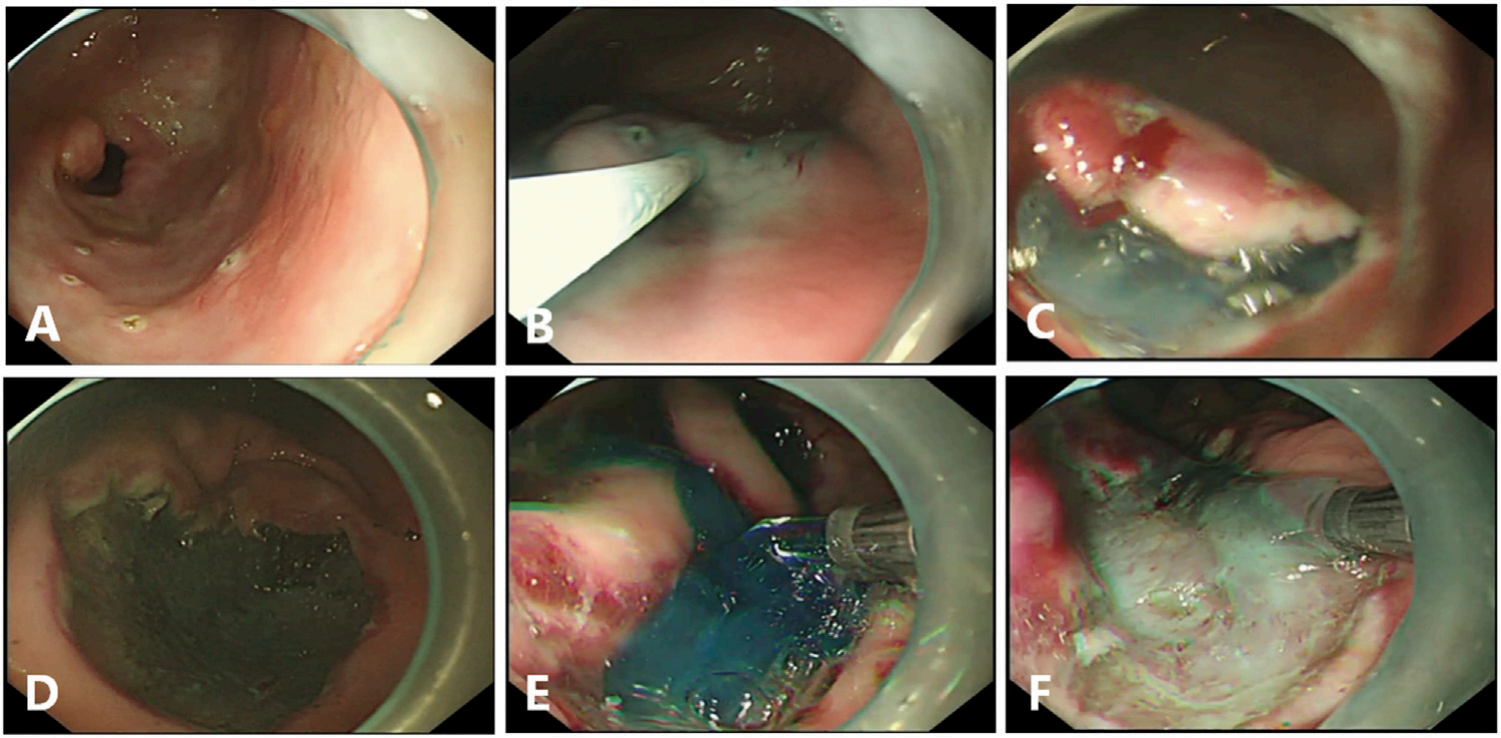
Endoscopic submucosal dissection (ESD) in pigs. **(A)**, Circumferential markings are made in a soft coagulation mode. **(B)**, The mixed solution is injected into the submucosal layer. **(C)**, Submucosal dissection is carried out by using forced coagulation mode. **(D)**, The Coagrasper forceps is used for major bleeding, by using soft coagulation mode. **(E, F)**, Gel application in ulcer bed immediately after the end of ESD with or without the dye.

A total of 16 lesions in five pigs were successfully achieved. The procedure time, injection time, resection rate, and cutting speed show no differences between the two groups (Table 1). Bleeding that could be considered significant rarely occurred. No perforation occurred during the procedure. After ESD, each ulcer bed in the gel-treated group was carefully coated with the novel gel (Figure 1). The volume of colloid and fixative solution used was 28.75 ± 5.18 and 18.13 ± 3.72 ml, separately. Total volume of gel used was 46.88 ± 8.84 ml. The time of colloid and fixative solution application was 24.25 ± 4.50 and 12.25 ± 1.98 s, separately. Total time of gel application was 36.50 ± 6.21 s.

**TABLE 1:** Details of the ESD procedures.

Variable	Control group	Gel-treated group	P value
Procedure time, mean ± SD, s	747 ± 588	603 ± 249	0.541
Injection time, mean ± SD, s	68 ± 54	69 ± 58	0.996
Long diameter of the specimen, mean ± SD, mm	21.2 ± 5.2	25.6 ± 4.2	0.102
Short diameter of the specimen, mean ± SD, mm	18.2 ± 4.0	21.8 ± 4.0	0.120
Lesion size, mean, mean ± SD, mm^2^	397.7 ± 184.7	571.0 ± 200.3	0.124
Cutting speed, mean ± SD, mm^2^/s	0.86 ± 0.54	1.00 ± 0.36	0.555
Major bleeding rate, n (%)	16.7	37.5	0.580
Perforation rate, n (%)	0	0	---

Two ulcer sites in different groups blended in one pig and the data of these ulcers were excluded.

**FIGURE 2:**
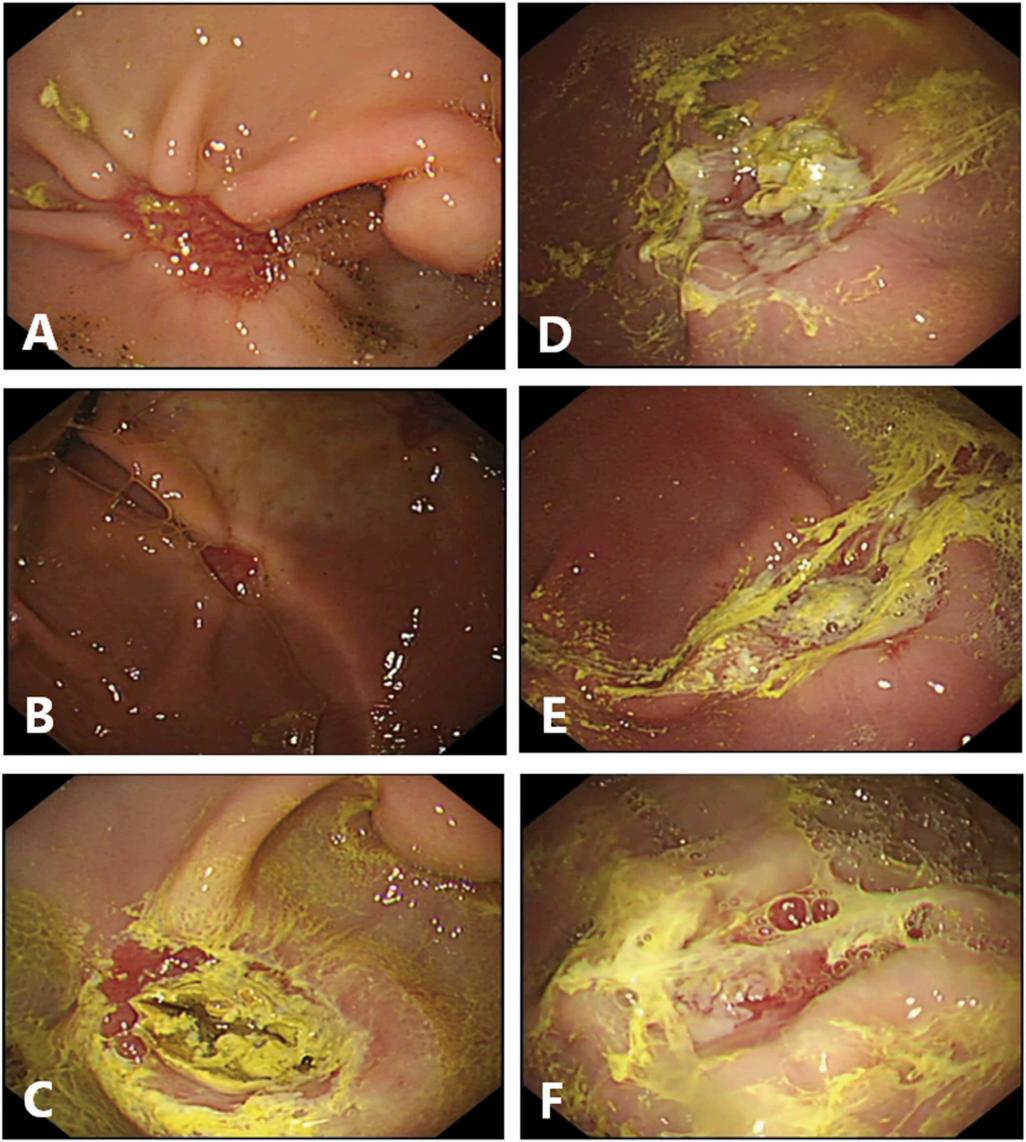
Endoscopic finding in the ulcer site on day 7 after endoscopic dissection. **(A–C)**, Endoscopic pictures in control group. The sizes of the artificial ulcers are reduced. **(C)**, Black crust existed at the ulcer floor in the pig present with hematemesis the day before endoscopic examination. **(D, F)**, Endoscopic pictures in gel-treated group. The sizes of the artificial ulcers are reduced too, and most of the gel still are attached, manifest as loosely white membranous film. **(D)**, Partially attached. **(E)**, fully attached. **(F)**, Lost.

**FIGURE 3:**
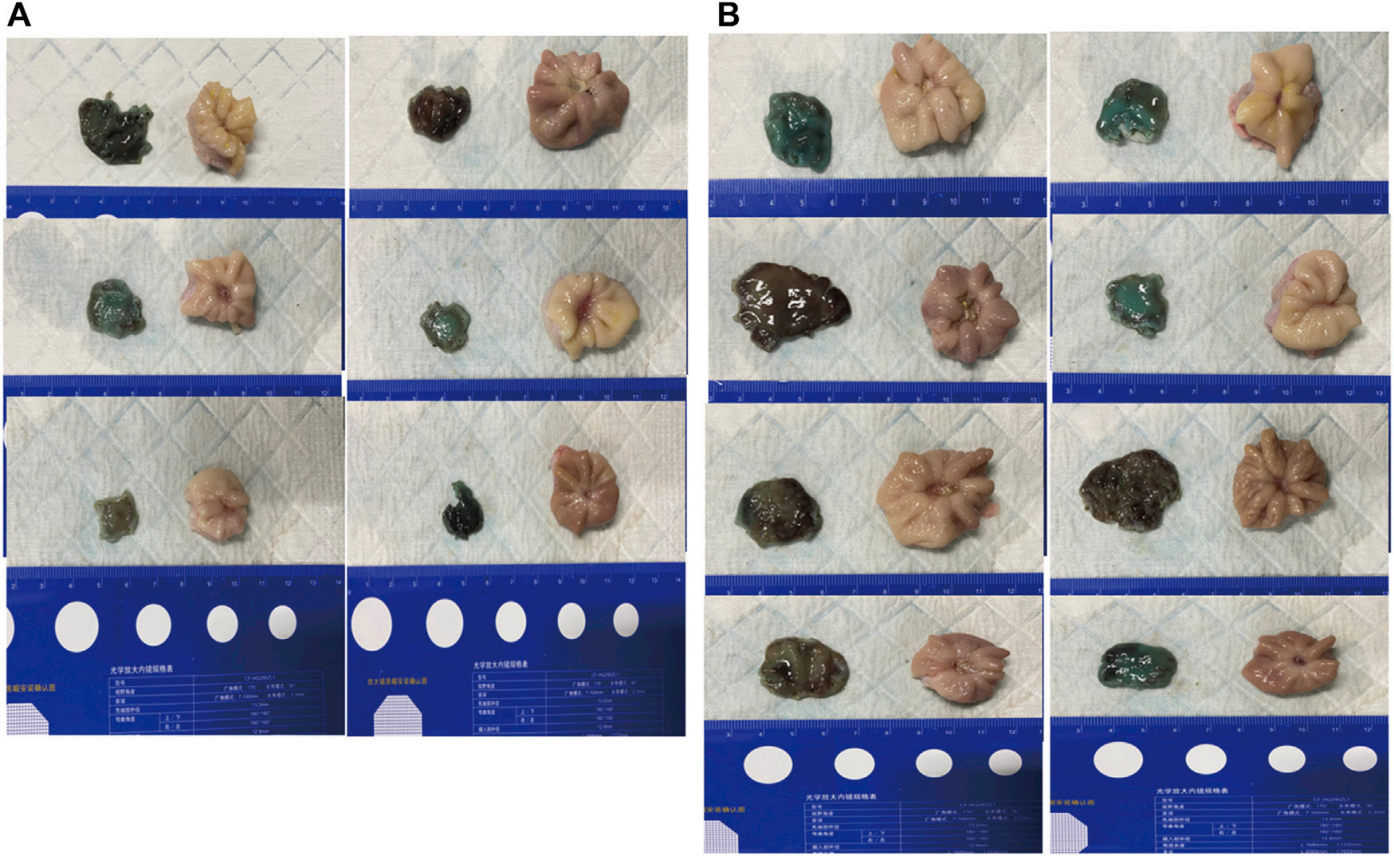
Gross photos for image analysis of the ratio of healed area in the remaining gastric bed tissue compared with the matched dissected gastricmucosa. Those on the left of each photo are endoscopic submucosal dissection (ESD) specimens, and those on the right of each photo are matchedspecimens from the harvested stomachs, on day 14 after the procedure. **(A)**, The control group, **(B)**, The gel-treated group.

On day 6, massive hematemesis occurred in one of the five pigs, and it resolved spontaneously. Confirmation endoscopy was performed on day 7 with all pigs under inhalation anesthetic. The sizes of the 16 remaining ulcers were reduced and tended to show wound contraction. A black crust was observed at one ulcer site in the pig with hematemesis (control group). No delayed perforation happened in all pigs. A residual congealing gel was seen at six of the eight gel-treated ulcers, manifesting as loosely white membranous film compared with a thin whitish fur at the base of the untreated ulcer (Figure 2). 14 days after ESD, all ulcers were significantly reduced in size, and no residual gel was detected at the treated sites (Figure 3). All pigs were euthanized 14 days after the procedure.

### Evaluation of Artificial Ulcer Healing

Two ulcer sites in different groups blended in one pig, and the data of these ulcers were excluded. The percentage of healing was higher in the gel-treated ulcers than in control ones (96.2 ± 2.2% vs. 91.9 ± 4.5%, *p* = 0.035) (Table 2).

**TABLE 2:** Comparison of the percent healing area among groups on day 14 after ESD procedures.

Ulcer #	Control group			Gel group		
	Area of initial ulcer	Area of remaining ulcer	% Healing area	Area of initial ulcer	Area of remaining ulcer	% Healing area
1	11,306	315	97.2	5,810	176	97.0
2	3,180	395	87.6	4,381	95	97.8
3	3,151	225	92.9	26,650	1929	92.8
4	2,215	313	85.9	4,351	137	96.9
5	4,136	337	91.9	7,176	501	93.0
6	2,839	110	96.1	8,838	142	98.4
7			-	9,358	374	96.0
8			-	6,291	143	97.8
Mean ± SD			91.9 ± 4.5		96.2 ± 2.2	
P					0.035	

Two ulcer sites in different groups blended in one pig and the data of these ulcers were excluded.

### Histopathologic Evaluation

**FIGURE 4:**
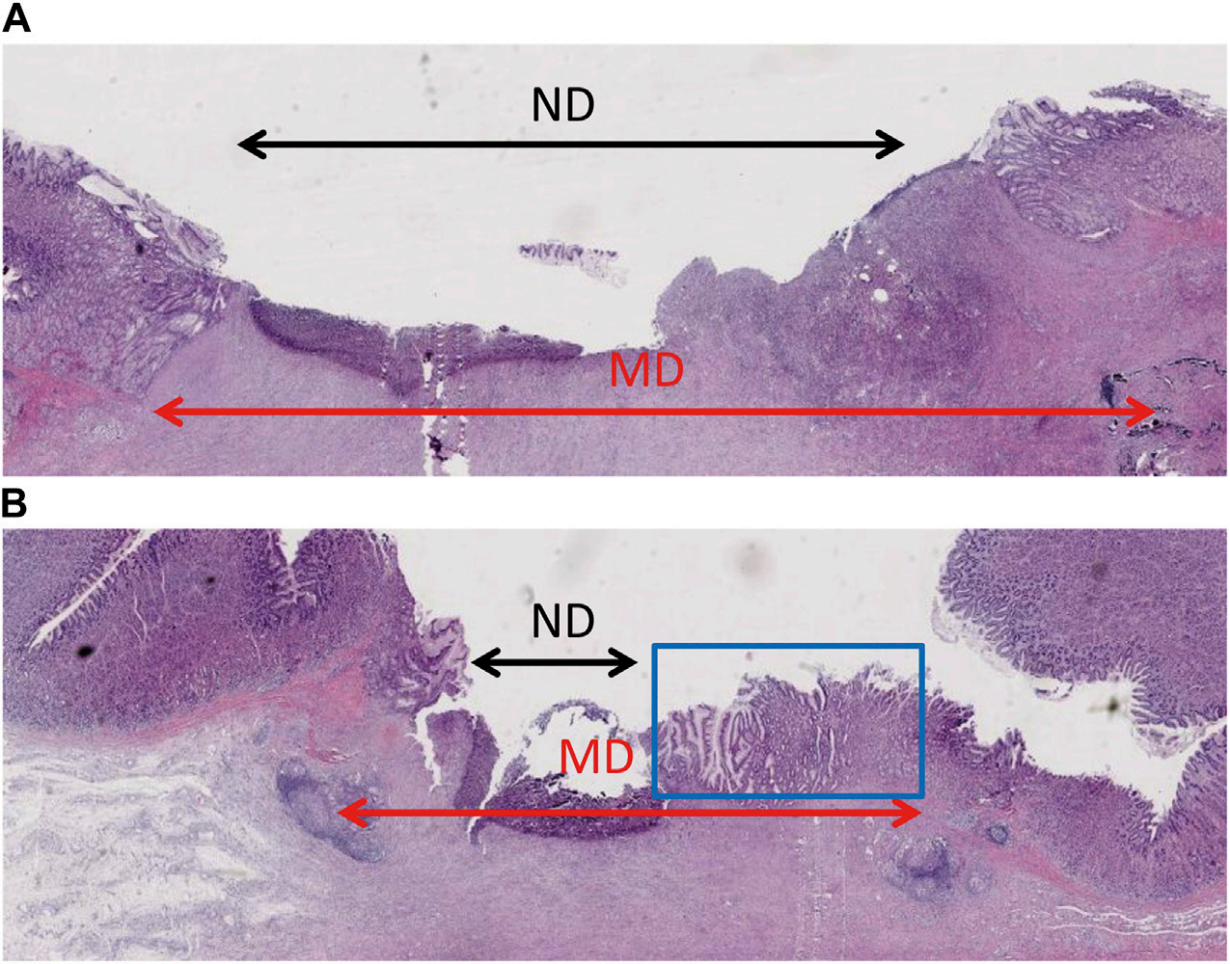
Histopathological examination showing epithelium regeneration. **(A)** The ulcer of the control group had a large non-epithelial covering diameter. **(B)** The non-epithelial covering area of the gel-coated group was relatively small, in which new epithelium can be seen in the blue box.

To determine the degree of epithelial regeneration of the ulcer tissue, the maximum diameter (ND) of the residual ulcer and the MD of the mucosal defect was measured. The percentage of MD after ND reduction was taken as the relative degree of epithelial regeneration of ulcers. In the control ulcers, the ND area was larger than in the gel group, which also showed a novel epithelium that covered the ulcer. The gel group showed higher epithelial regeneration than the control group (0.52 vs. 0.36, *p* = 0.031) (Figure 4; Table 3).

**TABLE 3:** Comparison of the degree of epithelial regeneration among groups on day 14 after ESD procedures.

Ulcer #	Control group			Gel group		
	ND, μm	MD, μm	Wre	ND, μm	MD, μm	Wre
1	9,721	13,434	0.28	4,501	10,583	0.57
2	9,915	13,894	0.29	4,929	7,963	0.38
3	1778	4,101	0.57	11,078	15,512	0.29
4	6,188	9,289	0.33	3,589	12,191	0.71
5	7,854	12,602	0.38	3,255	7,267	0.55
6	5,589	8,332	0.33	4,177	9,278	0.55
7			-	5,301	12,536	0.58
8			-	3,469	7,673	0.55
Mean ± SD			0.36 ± 0.11		0.52 ± 0.13	
P					0.031	

Two ulcer sites in different groups blended in one pig and the data of these ulcers were excluded.

## Discussion

ESD is a standard method for the management of early gastrointestinal neoplasia, but it results in large ulcers. Various methods have been tried to help the healing of ESD-induced artificial ulcers or to reduce the operative complication, but with unsatisfactory results. Therefore, this study aimed to examine the feasibility and effectiveness of a newly developed self-assembled gel on the ESD-induced ulcer healing process. The results suggest that the novel gel might promote the speed of ulcer healing after ESD, leading to higher epithelium formation. Further prospective clinical trials are needed to validate its effectiveness. This is the first randomized controlled study in animals that examined the feasibility and effectiveness of this novel self-assembled gel for the management of artificial ulcers after ESD.

In the early healing phase of artificial ulcers, it is critical to protect the ulcer floor from gastric or bile secretions. The material used in the present study is a self-assembled gel that solidifies in the presence of stomach acid. After ESD, each ulcer bed in the gel-treated group was successfully coated with the novel gel. On day 7 after ESD, compared with a thin whitish fur at the base of the untreated ulcers, a residual congealing gel was observed at six of eight treated ulcer sites, and this gel was almost gone on day 14. Therefore, most of the gel appears to remain for about 7 days, which is long enough to shield the ulcer from the gastric conditions, which are deleterious to proper healing, improving epithelialization and healing. Theoretically, the too long-lasting cover might impair the healing process. As reported, a bio-sheet grafts therapy used to cover ESD-induced artificial ulcer showed unexpected delayed ulcer healing (Kwon et al., 2015). In that study, the percentage of the attached bio-sheets was almost 81.8% (18/22) on day 7 after the procedure (Kwon et al., 2015). The authors pointed out that the membrane-like structure of the sheets might physically hinder the healing process of ulcers. In the present study, the residual gel detected by endoscopy was distributed loosely and flaky, resulting in no delayed healing.

Similar to this study, another study used a self-assembled peptide solution and showed promising results of helping decrease the risk of bleeding and accelerate the healing of the artificial gastric ulcers after ESD (Uraoka et al., 2016). The material used in that study was a fully-synthetic aqueous solution in which the monomers self-assemble at physiologic pH and forms a hydrogel made of a nanofibers. Hence, the gelatinous property of this material makes it relatively difficult to maintain full coverage of the ulcer floor because of gravity. On the other hand, the two components of the gel proposed here rapidly forms into a solid film just a few seconds once they are applied using the endoscope and become adherent to the ulcer floor because of the ionic features. The congealing property of the gel enables full and firm coverage of the ulcer floor, even during follow-up. Recently, other mechanical protective methods such as a polyglycolic acid sheet (PGAs) or bio-sheet had been reported to either accelerate the healing of artificial ulcers or to reduce the complications after ESD (Kwon et al., 2015; Sakaguchi et al., 2015). However, those strategies are very time consuming and causes maneuverability difficulties to some extent. As shown in our study, the total time of gel application under endoscopy was 36.50 ± 6.21 in gel group, which was much shorter than the reported time with other strategies like PGAs (Takao et al., 2015; Sakaguchi et al., 2021). A novel compound is also showing promising results but was not yet tested in animals (Chen et al., 2020).

This study has several limitations. First, although the difference in the healing rates between the two groups was statistically significant, the sample size was too small to reach firm conclusions. Second, despite the blue dye used to ensure the full coverage of gel for the ulcer bed, dye fading makes it a little difficult to distinguish a normal ulcer from a gel-treated one. Third, we used a standard catheter to deliver the gel, but we are currently developing a specific delivery catheter. This novel catheter is expected to achieve a more effective application of the material.

## Conclusion

In conclusion, this study strongly suggests the simplicity and practicality of the new gel. The use of this novel gel might provide shielding from the gastric conditions, as reflected in promoting the speed of ulcer healing, enhancing epithelialization. We expect this endoscopic treatment has clinical utility use after ESD. Further confirmation of the application and safety of this technique is necessary through clinical trials.

## Data Availability

The original contributions presented in the study are included in the article/Supplementary Material, further inquiries can be directed to the corresponding author.

## Abbreviations

ESD, endoscopic submucosal dissection; MD, mucosal dissected tissue diameter; ND, non-epithelized tissue diameter; PGAs, polyglycolic acid sheet.
